# Detecting Interval Distant Metastases With ^18^F-FDG PET/CT After Neoadjuvant Chemoradiotherapy for Locally Advanced Esophageal Cancer

**DOI:** 10.1097/RLU.0000000000004191

**Published:** 2022-04-05

**Authors:** Tiuri E. Kroese, Jelle P. Ruurda, Anne S. Bakker, Jasvir Jairam, Stella Mook, Sylvia van der Horst, Gert J. Meijer, Nadia Haj Mohammad, Peter S.N. van Rossum, Richard van Hillegersberg

**Affiliations:** From the Departments of ∗ Surgery; † Radiation Oncology; ‡ Medical Oncology, University Medical Center Utrecht, Utrecht University, Utrecht, the Netherlands.

**Keywords:** esophageal cancer, chemotherapy, radiotherapy, interval, metastases, ^18^F-FDG PET/CT

## Abstract

Patients with esophageal cancer can develop distant metastases between the start of neoadjuvant chemoradiotherapy (nCRT) and planned surgery (ie, interval distant metastases). ^18^F-FDG PET/CT restaging after nCRT detects interval distant metastases in ~8% of patients. This study aimed to identify patients for whom ^18^F-FDG PET/CT restaging after nCRT could be omitted using an existing prediction model predicting for interval distant metastases or by using clinical stage groups.

**Patients and Methods:**

Patients with locally advanced esophageal cancer who underwent baseline and restaging ^18^F-FDG PET/CT, nCRT, and were planned for esophagectomy between 2017 and 2021 were eligible for inclusion in this retrospective study. The primary outcome was the existing model’s external performance (ie, discrimination and calibration) for predicting interval distant metastases. The existing model predictors included tumor length, cN status, squamous cell carcinoma histology, and baseline SUV_max_. The secondary outcome determined the clinical stage groups (AJCC/UICC eighth edition) for adenocarcinoma and squamous cell carcinoma for which the incidence of interval distant metastases was <10%.

**Results:**

In total, 127 patients were included, of whom 17 patients developed interval distant metastases (13%; 95% confidence interval [CI], 8%–21%) and 9 patients were deemed to have false-positive lesions on ^18^F-FDG PET/CT (7%; 95% CI, 2%–11%). Applying the existing model to this cohort yielded a discriminatory c-statistic of 0.56 (95% CI, 0.40–0.72). The calibration of the existing model was poor (ie, mostly underestimating the actual risk). The incidence of true-positive versus false-positive interval distant metastases for patients with clinical stage II disease was 5% versus 0%; clinical stage III, 14% versus 8%; and clinical stage IVa, 22% versus 9%.

**Conclusions:**

The existing prediction model cannot reliably identify patients at risk for developing interval distant metastases after nCRT for esophageal cancer. Omission of ^18^F-FDG PET/CT restaging after nCRT could be considered in patients with clinical stage II esophageal cancer.

Esophageal cancer is the seventh most common cancer worldwide, with an estimated 572,000 new cases annually.^1^ A standard treatment for patients with locally advanced esophageal cancer consists of neoadjuvant chemoradiotherapy (nCRT) followed by esophagectomy.^2,3^ The most common nCRT regimen in Europe is weekly carboplatin and paclitaxel with a concurrent planned total radiation dose of 41.4 Gy in 23 fractions of 1.8 Gy (ie, CROSS).^4^ Distant metastases can develop between the start of nCRT and surgery, so-called interval distant metastases. Restaging after nCRT with ^18^F-FDG PET/CT detects interval distant metastases in approximately 8% of patients.^5,6^ For these patients, a noncurative esophagectomy and the associated complications can be prevented. However, in approximately 92% of patients, ^18^F-FDG PET/CT restaging does not detect interval distant metastases, and restaging has no impact on treatment decision-making.^6^ For these patients, restaging only increases patient anxiety and hospital costs. In addition, the false-positive rate of ^18^F-FDG PET/CT restaging after nCRT is 5% to 6%.^5,6^ False-positive lesions and subsequent additional biopsies can cause profound psychosocial harm to the patient.^7^

An individualized approach of restaging would identify patients at risk for developing interval distant metastases and reduce unnecessary testing. Thus, a model for predicting interval distant metastases after CRT for locally advanced esophageal cancer was developed and internally validated at the MD Anderson Cancer Center.^6^ In that study, a model with satisfactory model performance was developed and internally validated (adjusted c-statistic, 0.67).^6^ However, the applicability of this model in the setting of the CROSS regimen remains unclear because no external validation has been performed so far. Moreover, the model was developed in patients treated with neoadjuvant or definitive CRT to a dose of 50.4 Gy (rather than 41.4 Gy) and mainly fluoropyrimidine-based chemotherapy (rather than carboplatin/paclitaxel).^4^ Another individualized approach to restaging would be to perform restaging after nCRT for patients with a more advanced clinical stage (eg, III–IVa). The AJCC/UICC eighth edition produced recommendations for the clinical stage groups of esophageal and esophagogastric junction cancer patients.^8^ This recommendation was based on the Worldwide Esophageal Cancer Collaboration data, including 22,123 clinically staged patients from 33 institutions.^9^ The clinical stage groups for adenocarcinoma and squamous cell carcinoma were associated with overall survival and with pathological stage groups.^9^

This retrospective cohort study aimed to identify patients for whom ^18^F-FDG PET/CT restaging after nCRT for locally advanced esophageal cancer could be omitted using an existing model predicting interval distant metastases or by using clinical stage groups (AJCC/UICC eighth edition) for adenocarcinoma and squamous cell carcinoma.

## PATIENTS AND METHODS

### Study Design

The institutional review board approved this study, and the need for informed consent was waived. The study was performed in accordance with the ethical standards as laid down in the 1964 Declaration of Helsinki and its later amendments, and was reported according to the guidelines of the Transparent Reporting of a Multivariable Prediction Model for Individual Prognosis or Diagnosis (Supplementary File A, Supplemental Digital Content 1, http://links.lww.com/CNM/A374).^10^

### Patient Inclusion

Consecutive patients with locally advanced esophageal cancer who received nCRT according to the CROSS protocol and were scheduled for a transthoracic or transhiatal esophagectomy with gastric tube reconstruction between January 2017 and April 2021 at the UMC Utrecht were eligible for inclusion in this retrospective study. All patients underwent step-and-shoot intensity-modulated radiotherapy or volumetric modulated arc therapy. The total planned radiation dose of 41.4 Gy was given in 23 fractions of 1.8 Gy, with 5 fractions administered per week, starting on the first day of the first chemotherapy cycle.^4^ Carboplatin was targeted at an area under the curve of 2 mg/mL per minute and paclitaxel at a dose of 50 mg per square meter of body surface area, and both were administered intravenously in 5 cycles.^4^ Patients with unresectable (cT4b) or metastatic disease (cM1) at baseline or who were planned for definitive chemoradiotherapy (ie, 50.4 Gy) were not eligible for inclusion. Patients without routine ^18^F-FDG PET/CT restaging after nCRT or with a time interval between completing nCRT and ^18^F-FDG PET/CT restaging of >3 months were excluded.

### Image Acquisition and Staging

All ^18^F-FDG PET/CT imaging was performed on an EARL-accredited system.^11^ Patients were instructed to fast at least 6 hours before injection of the ^18^F-FDG contrast agent (targeted at 2.0 MBq/kg), and glucose level within the reference range (80–120 mg/dL) was confirmed. A CT scan without contrast agent was acquired for attenuation correction purposes. ^18^F-FDG PET image acquisition was performed 60 to 90 minutes after ^18^F-FDG injection in 3-dimensional acquisition mode at 2 to 5 minutes per bed position. Restaging after nCRT was routinely performed in patients with locally advanced esophageal cancer with a more advanced clinical stage (ie, III–IVa) and in patients with any locally advanced disease stage included in a prospective response assessment trial.^12,13^ The AJCC/UICC eighth edition was used for clinical and pathological staging.^8^ The ^18^F-FDG PET/CT scans were reviewed by experienced nuclear medicine physicians and discussed in a multidisciplinary tumor board meeting with dedicated upper gastrointestinal surgeons, gastroenterologists, pathologists, medical oncologists, radiation oncologists, and radiologists.

### Interval Distant Metastases

Interval distant metastases were defined as new distant lesions (according to the AJCC/UICC eighth edition) detected by ^18^F-FDG PET/CT restaging after nCRT, before planned surgery.^8^ True-positive lesions were lesions with pathologic evidence of malignancy (ie, histology or cytology) or, if tissue could not be obtained, were confirmed with repeated follow-up imaging. False-positive lesions were lesions with pathologic evidence of nonmalignancy or, if tissue could not be obtained, were no longer suspicious on repeated follow-up imaging. False-negative lesions were lesions detected during planned surgery, which were not visible on ^18^F-FDG PET/CT restaging after nCRT and with pathologic evidence of malignancy.

### Primary Outcome

The primary outcome was the existing model’s external performance (ie, discrimination and calibration). Predictors of the existing model constructed for interval distant metastases were baseline clinical nodal stage (cN+ vs cN0), endoscopic ultrasound (EUS)–based tumor length (≥4.0 cm vs <4.0 cm), tumor histology (squamous cell carcinoma vs adenocarcinoma), and baseline SUV_max_ of the primary tumor (≥9.6 vs <9.6).^6^ The tumor histology was determined on the pretreatment biopsy of the primary tumor. The SUV_max_ of the primary tumor was defined as the SUV_max_ of the gross tumor volume and was extracted using Volumetool.^14^ A risk score was calculated for each clinical predictor (2 points for cN+ stage and EUS-based tumor length ≥4 cm, and 1 point for squamous cell carcinoma histology and SUV_max_ ≥9.6). The total risk score was obtained by adding up the number of points for each clinical predictor. The existing prediction model logistic regression formula was as follows:


$$\;\log\left(\frac{p}{1-p}\right)=-4.425+0.940\times \mathrm{cN}\;\text{stage}+0.869\times \mathrm{EUS}- -\text{based tumor length}+0.440\times \text{tumor histology}+0.448\times \text{SUVmax}.$$

### Secondary Outcome

The secondary analysis determined the clinical stage groups for which the incidence of interval distant metastases was <10% among ≥10 patients. The cutoff of 10 patients was used because the expected incidence of interval metastases was 8%, and therefore at least 1 of 10 patients was expected to develop an interval distant metastases.^5^ Clinical stage groups were defined at baseline (ie, before nCRT) according to the AJCC/UICC eighth edition.^9^

### Statistical Analysis

Continuous normally distributed variables were expressed using mean with standard deviation (SD) and compared using Student’s *t* test. Continuous nonparametric data were expressed using median with interquartile range (IQR) and compared using Mann-Whitney *U* test. Categorical variables were expressed using frequencies with percentages and compared using Fisher’s exact test. The discriminative performance of the model was assessed using the c-statistic and was illustrated with a receiver operating characteristic curve. The model calibration was evaluated comparing the observed risk of interval distant metastases with 3 equal size predicted risk groups using the existing prediction model. There were no missing data in predictor or outcome parameters of the model. All statistical analyses were performed using R version 3.5.1 (packages “rms” and “ggplot2”). A *P* value of <0.05 was considered statistically significant.

## RESULTS

**FIGURE 1:**
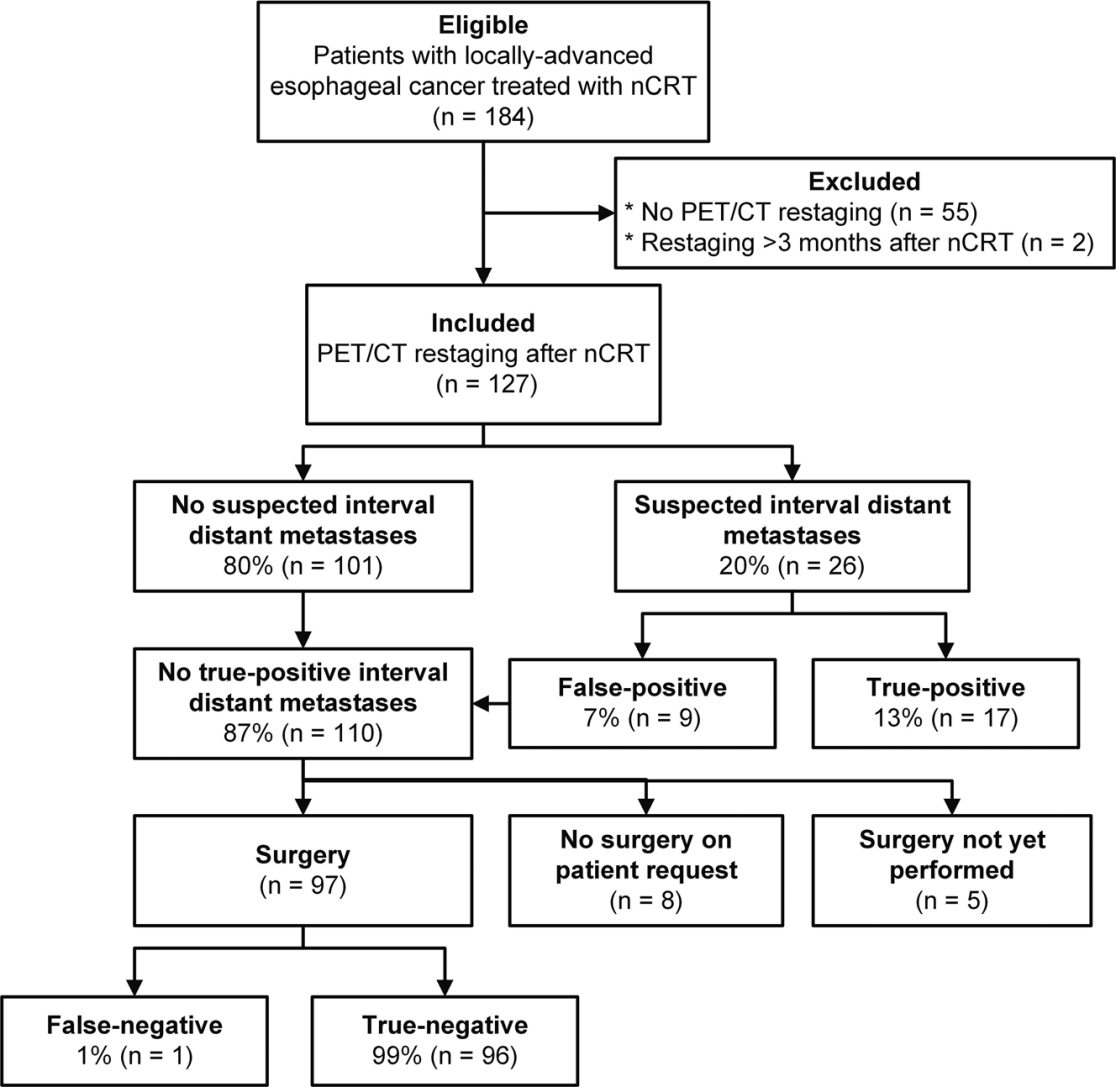
Patient inclusion.

A total of 184 patients who underwent baseline ^18^F-FDG PET/CT imaging and nCRT for locally advanced esophageal cancer were assessed for eligibility, of whom 55 patients were excluded because they did not undergo ^18^F-FDG PET/CT restaging after nCRT and 2 patients because ^18^F-FDG PET/CT restaging was performed >3 months after completion of nCRT. Consequently, 127 patients were included. Figure 1 shows the patient inclusion.

Patients were mostly male (76%), with a mean age of 66 years (SD ± 9.5) and a mean BMI of 26 (SD ± 4.5). The primary tumor was predominantly an adenocarcinoma (76%), located in the distal esophagus (90%). The most common clinical tumor stage was T3 (80%), and the clinical nodal stage N1 (45%). The median time interval between completing nCRT and ^18^F-FDG PET/CT restaging was for patients with versus without interval distant metastases: 43 days (IQR, 39–53) versus 47 days (IQR, 40–54). For patients without interval distant metastases, the median time interval between ^18^F-FDG PET/CT restaging and surgery was 39 days (IQR, 28–49), and the total median time interval between completing nCRT and surgery was 82 days (IQR, 72–92). Table 1 demonstrates the patient characteristics.

**TABLE 1:** Baseline Characteristics

Characteristic	Interval Distant Metastases		*P*
	No (n = 110)	Yes* (n = 17)	
Sex (%)			0.917
Male	82 (75.0)	14 (82.4)	
Female	28 (25.0)	3 (17.6)	
Mean age in years (±SD)	65.6 (±9.5)	68.6 (±9.7)	0.355
Mean body mass index (±SD)	26.1 (4.3)	25.1 (5.5)	0.297
Differentiation grade			0.001
Well	9 (8.3)	0 (0.0)	
Moderate	44 (40.7)	8 (47.1)	
Poor	46 (42.6)	2 (11.8)	
Missing	1 (0.9)	7 (41.1)	
Tumor histology (%)†			1.000
Adenocarcinoma	84 (75.9)	13 (76.5)	
Squamous cell carcinoma	26 (22.2)	4 (23.5)	
Tumor location (%)			0.289
Proximal third esophagus	1 (0.9)	1 (6.2)	
Middle third esophagus	8 (7.4)	0 (0.0)	
Distal third esophagus	101 (91.7)	16 (94.1)	
Median tumor length in cm [IQR]‡	5.0 [3.0, 6.0]	5.0 [4.0, 6.0]	0.656
Clinical tumor stage (%)§			0.509
T1b	2 (1.8)	0 (0.0)	
T2	16 (13.0)	2 (11.8)	
T3	87 (80.6)	13 (76.5)	
T4a	5 (4.6)	2 (11.8)	
Clinical nodal stage (%)§			0.624
N0	41 (36.1)	5 (29.4)	
N1	48 (43.5)	6 (35.3)	
N2	15 (13.9)	3 (17.6)	
N3	6 (5.6)	3 (17.6)	
Clinical stage group (%)§			
Stage I	2 (0.0)	0 (0.0)	0.563
Stage II	19 (17.3)	1 (5.9)	
Stage III	73 (66.4)	12 (70.6)	
Stage IVa	17 (15.5)	4 (23.5)	
Median SUV_max_ primary tumor [IQR]¶	12.6 [9.7, 17.1]	15.6 [10.7, 17.9]	0.486
Staging modalities			
Endoscopy	110 (100%)	17 (100%)	
Baseline ^18^F-FDG PET/CT	110 (100%)	17 (100%)	
Restaging ^18^F-FDG PET/CT	110 (100%)	17 (100%)	
Median time interval in days [IQR]			0.186
nCRT and restaging	43 [39–53]	47 [40–54]	
Restaging and surgery	39 [28–49]	NA	
nCRT and surgery	82 [72–92]	NA	
Radiotherapy technique (%)			0.284
Intensity modulated radiotherapy	1 (0.9)	1 (5.9)	
Volumetric modulated arc therapy	109 (99.1)	16 (94.1)	

*True-positive.

†Determined on pretreatment biopsy.

‡Determined on EUS.

§AJCC/UICC eighth edition.

¶Determined on the gross tumor volume of the primary tumor at baseline.

Interval distant metastases were detected by ^18^F-FDG PET/CT restaging in 17 patients (13%; 95% confidence interval [CI], 8%–21%) (Fig. 3). Interval distant metastases were confirmed with a histological biopsy (n = 14) or on repeated follow-up imaging in case tissue could not be acquired (n = 3). False-positive distant lesions were detected by ^18^F-FDG PET/CT restaging in 9 patients (7%; 95% CI, 3%–13%) (Fig. 4). Lesions were confirmed to be not malignant with a histological biopsy (n = 5) or on repeated follow-up imaging in case tissue could not be obtained (n = 4). In total, 97 patients (76%) underwent surgery after nCRT. Distant metastases not detected by ^18^F-FDG PET/CT restaging (ie, false-negative) were detected during planned surgery in 1 patient (1%; 95% CI, 0%–6%). The liver metastasis in this patient was confirmed with a histological biopsy. Table 2 demonstrates the characteristics of true-positive, false-positive, and false-negative interval distant metastases.

**TABLE 2:** Characteristics of True-Positive, False-Positive, and False-Negative Interval Distant Metastases

	Interval Distant Metastases					
	True-Positive		False-Positive		False-Negative	
Location	(n = 17)		(n = 9)		(n = 1)	
Extraregional lymph node	5	29%	4	44%	—	—
Bone	4	24%	—	—	—	—
Liver	3	18%	—	—	1	100%
Adrenal gland	2	12%	—	—	—	—
Lung	1	6%	4	44%	—	—
Multiple locations	2	12%	1	11%	—	—
No. lesions						
1	6	35%	5	55%	—	—
2	1	6%	3	33%	—	—
3	3	18%	1	11%	—	—
>3	7	41%	—	—	1	100%
Reference standard						
Histological biopsy	14	82%	5	66%	1	100%
Repeated follow-up imaging	3	18%	4	44%	—	—

**FIGURE 2:**
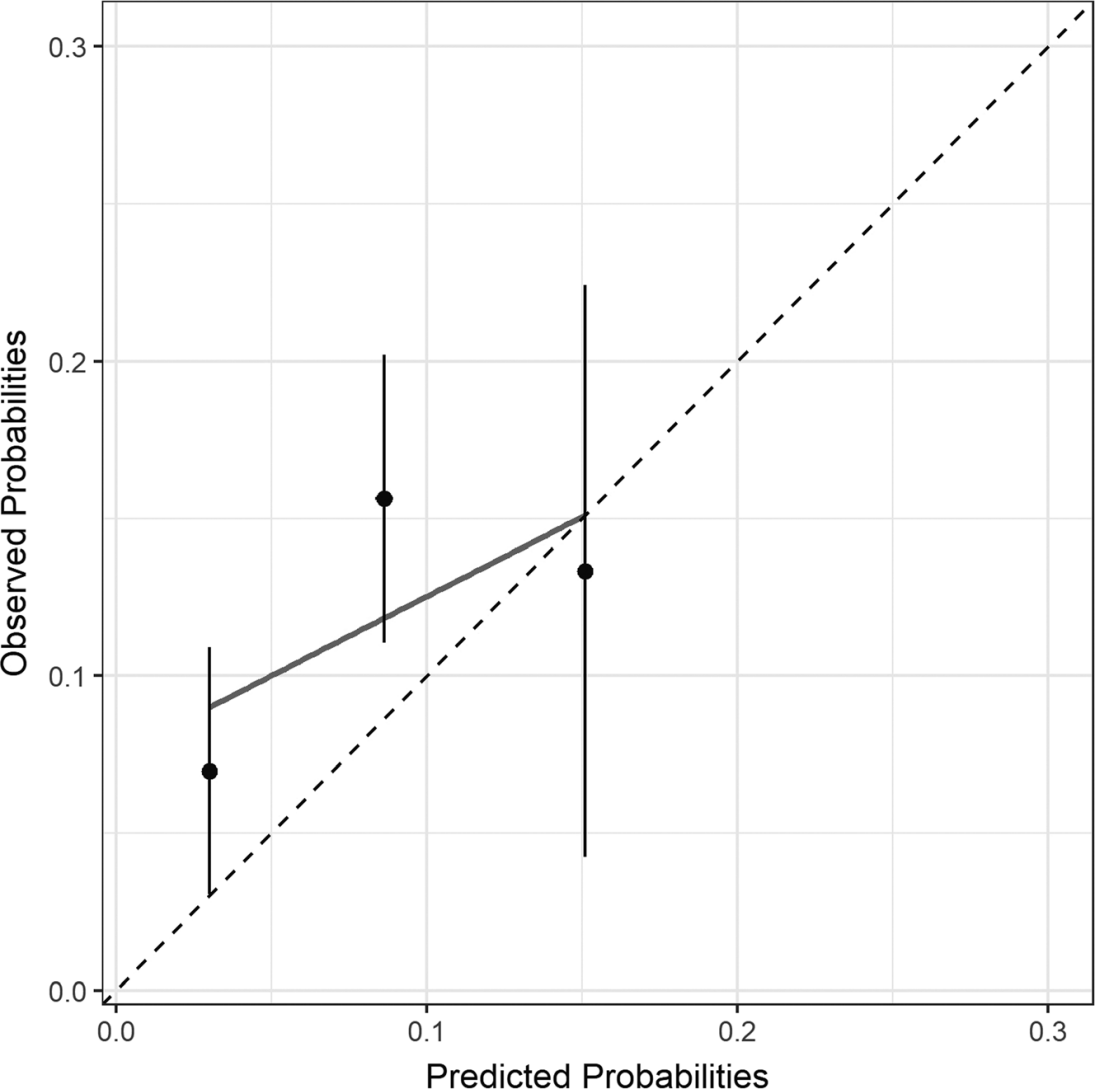
Calibration of the existing model predicting interval distant metastases in this external cohort.

The cN stage, EUS-based tumor length, tumor histology, and baseline SUV_max_ of the primary tumor were comparable between patients with and without interval distant metastases (*P* = 0.721, *P* = 0.885, *P* = 1.000, and *P* = 0.876, respectively; Table 3). The total risk score of the existing model was not associated with interval distant metastases (*P* = 0.080). Applying the existing model to this external cohort yielded a discriminatory c-statistic of 0.56 (95% CI, 0.40–0.72). The existing model discrimination is presented in Supplementary File B, Supplemental Digital Content 2, http://links.lww.com/CNM/A375. Calibration of the existing model in this external cohort was poor. Overall, the model mainly underestimated the actual risk of interval distant metastases (Fig. 2).

**TABLE 3:** Association Between Predictors of the Existing Prediction Model and Interval Distant Metastases

	Interval Distant Metastases			
Predictor	No (n = 110)		Yes* (n = 17)		*P*
Clinical nodal stage†					0.721
cN0	41	37%	5	29%	
cN+ (2 points)	69	63%	12	71%	
EUS-based tumor length in cm					0.885
<4.0	36	33%	5	29%	
≥4.0 (2 points)	69	67%	12	71%	
Missing	5	5%	0	0%	
Tumor histology‡					1.000
Adenocarcinoma	84	76%	13	76%	
Squamous cell carcinoma (1 point)	26	24%	4	24%	
Baseline SUV_max_ primary tumor§					0.876
<9.6	25	23%	3	18%	
≥9.6 (1 point)	85	77%	14	82%	
No. points					0.080
0	3	3%	2	12%	
1	11	10%	1	6%	
2	16	14%	2	12%	
3	24	22%	0	0%	
4	11	10%	1	6%	
5	32	29%	9	53%	
6	13	11%	2	12%	

*True-positive.

†Determined on pretreatment biopsy.

‡AJCC/UICC eighth edition.

§Determined on the gross tumor volume of the primary tumor.

**FIGURE 3:**
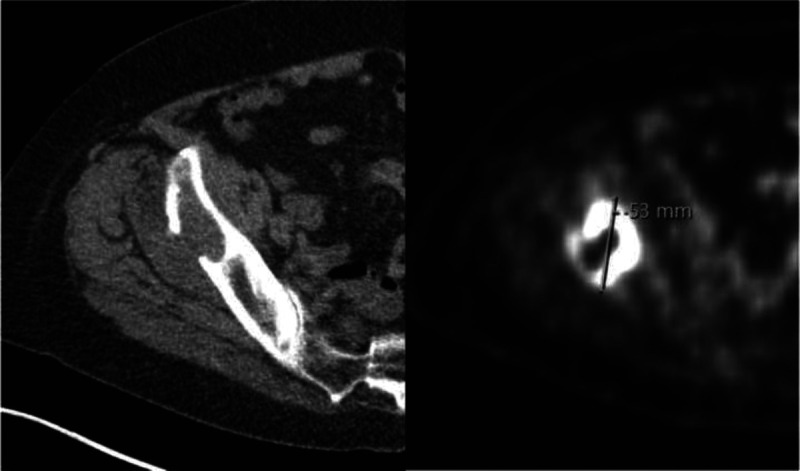
Example of a true-positive lesion detected by ^18^F-FDG PET/CT restaging. A 78-year-old woman with a cT3N0M0 mid-esophageal squamous cell carcinoma treated with neoadjuvant chemoradiation with a pathologic lytic fracture of the right ileum with pathologic PET activity. A histological biopsy showed a squamous cell carcinoma.

**FIGURE 4:**
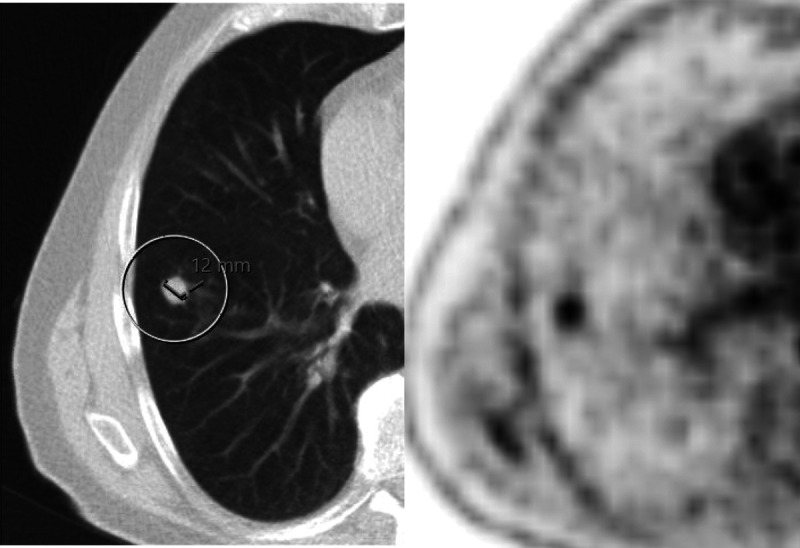
Example of a false-positive lesion detected by ^18^F-FDG PET/CT restaging. A 69-year-old man with a cT3N1M0 distal-esophageal adenocarcinoma treated with neoadjuvant chemoradiation with a 12-mm nodule in the right lower lobe with pathologic PET activity. A histological biopsy showed fibrosis, which was confirmed with follow-up imaging.

Subsequently, the AJCC/UICC baseline (ie, before nCRT) clinical-staging groups for adenocarcinoma and squamous cell carcinoma were used to determine the incidence of interval distant metastases. The incidence of true-positive versus false-positive interval distant metastases for initial clinical stage II disease was 5% (1/20) versus 0% (0/20) in patients with clinical stage III disease 14% (12/85) versus 8% (7/85), and 19% (4/21) versus 10% (2/21) in patients with clinical stage IVa disease. The incidence of true-positive versus false-positive interval lesions detected by ^18^F-FDG PET/CT per stage group for adenocarcinoma, squamous cell carcinoma, and combined is presented in Table 4 (Figs. 3, 4).

**TABLE 4:** Rate of True-Positive Versus False-Positive Interval Distant Metastases Per Clinical Stage Group and Histology

	Adenocarcinoma					Squamous Cell Carcinoma					Combined			
Stage Group*	cTN Stage*	True-Positive†		False-Positive†		cTN Stage*	True-Positive†		False-Positive†		True-Positive†		False-Positive†	
I	T1N0	—	0/0	—	0/0	T1N0–1	—	0/2	—	0/2	0/2	—	0/2	—
II	T1N1, T2N0	0%	0/10	0%	0/10	T2N0–1, T3N0	10%	1/10	0%	0/10	1/20	5%	0/20	0%
III	T2N1, T3-4aN0–1	14%	10/72	7%	5/72	T3N1, T1-3N2	15%	2/13	15%	2/13	12/85	14%	7/85	8%
IVa	T1-4aN2–3	19%	3/16	6%	1/16	T1-4aN3, T4aN0–2	20%	1/5	20%	1/5	4/21	19%	2/21	10%

*AJCC/UICC eighth edition.

†Interval distant metastases.

cTN, clinical tumor and nodal stage.

## DISCUSSION

This study included 127 patients with locally advanced esophageal cancer who underwent routine baseline and restaging ^18^F-FDG PET/CT imaging and nCRT according to the CROSS regimen. External validation of an existing prediction model predicting for interval distant metastases yielded a poor discriminative performance (c-statistic, 0.56) and poor calibration (ie, predominantly underestimating the actual risk of interval distant metastases). Thus, the existing model does not reliable identify individual patients at risk for developing interval distant metastases. Based on AJCC/UICC clinical stage groups, omission of ^18^F-FDG PET/CT restaging after nCRT in patients with clinical stage II disease can be considered because the risk of interval distant metastases was 5% (below our prespecified threshold of 10%). Thus, we recommend restaging with ^18^F-FDG PET/CT after nCRT in patients with stage ≥III disease of the esophagus or gastroesophageal junction.

The poor external performance of the existing prediction model might be explained by differences in treatment between this cohort and the cohort used for model development.^6^ The patients included in our cohort received a lower planned radiotherapy dose (41.4 Gy vs 50.4 Gy), a different type of chemotherapy (carboplatin/paclitaxel vs mainly fluoropyrimidine), and a lower number of chemotherapy cycles (4 vs 5) as compared with the cohort used for model development.^6^ However, the clinical tumor stage (cT1b–T2), clinical nodal stage (cN0), and tumor differentiation grade (well or moderate) were comparable between our cohort and the cohort used for model development (16% vs 12%, 36% vs 34%, and 48% vs 46%, respectively).^6^ The higher incidence of interval distant metastases among patients with adenocarcinoma histology in our cohort (13%, 13/97) as compared with the cohort used for model development (7.6%, 51/672, *P* < 0.001)^6^ supports the interpretation that nCRT might not be the optimal neoadjuvant treatment for all patients.^15^ Accordingly, NCCN and ESMO guidelines recommend either nCRT or perioperative chemotherapy (eg, FLOT^16^) for patients with esophageal adenocarcinoma.^2,3^

This study shows that higher clinical stage groups were associated with an increased risk of interval distant metastases (ie, 5% for stage II, 14% for stage III, and 22% for stage IVa). The relatively low risk of interval distant metastases in patients with clinical stage II disease might be explained by the less aggressive tumor biology as compared with patients with stage III to IV disease. Accordingly, ESMO and NCCN guidelines recommend upfront resection for patients with stage II disease and as compared with nCRT followed by resection in patients with stage III to IVa disease.^2,3^ However, understaging of the actual pathological nodal disease occurs in 27% to 56% of patients (ie, cN0 and ypN+).^17–20^ Accordingly, some studies have shown improved overall survival in patients with stage II disease treated with nCRT followed by esophagectomy compared with upfront esophagectomy alone.^21,22^ No recommendation on restaging for patients with stage I disease can be made because 2 patients only were included with stage I disease (which was below our prespecified threshold of at least 10 patients).

Strength of this study includes the homogeneity of the study cohort since only patients with locally advanced esophageal cancer undergoing baseline and restaging ^18^F-FDG PET/CT and nCRT according to the CROSS protocol were included. Other strengths include subgroup analyses for adenocarcinoma and squamous cell carcinoma. This study also contains certain limitations that need to be taken into consideration when interpreting the results. First, the relatively limited sample size for smaller clinical stage groups (ie, I, II, or IVa) may have caused both an overestimation and underestimation of the actual rate of true-positive and false-positive interval distant metastases. Second, no new model for the development interval distant metastases could be constructed because of the limited number of events. Third, 55 patients had to be excluded from this study because these patients did not receive restaging after nCRT either because they did not want to participate in a response assessment trial or did not have a locally advanced disease stage. Therefore, the proportion of patients with stage I to II disease could be underrepresented in our cohort.

In conclusion, external validation of an existing model yielded poor discrimination (c-statistic, 0.56) and poor calibration (ie, predominantly underestimating actual incidence of interval distant metastases). Thus, this model cannot reliably identify individual patients at risk for developing interval distant metastases after nCRT. Based on AJCC/UICC clinical-staging groups, omission of ^18^F-FDG PET/CT restaging after nCRT could be considered in patients with clinical stage II disease (ie, cT2N0 or cT1N1 adenocarcinoma and T2N0–1 or T3N0 squamous cell carcinoma) because the rate of interval distant metastases was 5% (i.e., below our prespecified threshold of 10%). We recommend routine restaging with ^18^F-FDG PET/CT after nCRT for stage ≥III disease.
